# Edelfosine nanoemulsions inhibit tumor growth of triple negative breast cancer in zebrafish xenograft model

**DOI:** 10.1038/s41598-021-87968-4

**Published:** 2021-05-10

**Authors:** Sofia M. Saraiva, Carlha Gutiérrez-Lovera, Jeannette Martínez-Val, Sainza Lores, Belén L. Bouzo, Sandra Díez-Villares, Sandra Alijas, Alba Pensado-López, Abi Judit Vázquez-Ríos, Laura Sánchez, María de la Fuente

**Affiliations:** 1 Nano-Oncology and Translational Therapeutics Unit, Health Research Institute of Santiago de Compostela (IDIS), Clinical University Hospital of Santiago de Compostela (CHUS), SERGAS, Santiago de Compostela, Spain; 2 Department of Zoology, Genetics and Physical Anthropology, Campus of Lugo, University of Santiago de Compostela, Lugo, Spain; 3 Cancer Network Research (CIBERONC), Madrid, Spain; 4 Center for Research in Molecular Medicine and Chronic Diseases (CiMUS), University of Santiago de Compostela, Santiago de Compostela, Spain

**Keywords:** Cancer, Drug discovery, Nanoscience and technology

## Abstract

Triple negative breast cancer (TNBC) is known for being very aggressive, heterogeneous and highly metastatic. The standard of care treatment is still chemotherapy, with adjacent toxicity and low efficacy, highlighting the need for alternative and more effective therapeutic strategies. Edelfosine, an alkyl-lysophospholipid, has proved to be a promising therapy for several cancer types, upon delivery in lipid nanoparticles. Therefore, the objective of this work was to explore the potential of edelfosine for the treatment of TNBC. Edelfosine nanoemulsions (ET-NEs) composed by edelfosine, Miglyol 812 and phosphatidylcholine as excipients, due to their good safety profile, presented an average size of about 120 nm and a neutral zeta potential, and were stable in biorelevant media. The ability of ET-NEs to interrupt tumor growth in TNBC was demonstrated both in vitro, using a highly aggressive and invasive TNBC cell line, and in vivo, using zebrafish embryos. Importantly, ET-NEs were able to penetrate through the skin barrier of MDA-MB 231 xenografted zebrafish embryos, into the yolk sac, leading to an effective decrease of highly aggressive and invasive tumoral cells’ proliferation. Altogether the results demonstrate the potential of ET-NEs for the development of new therapeutic approaches for TNBC.

## Introduction

Cancer is one of the major health problems worldwide due to its high rate of morbidity and mortality. Among the different types of cancer, breast cancer is the second most prevalent, with an estimation of two million new cases in 2018, and is the leading cause of death in women worldwide^1^. It represents a heterogeneous group of tumors that is currently classified in five different subtypes according to their histological and molecular patterns^2^. Among these subtypes, TNBC accounts for 15–20% of the cases. TNBC lacks of the expression of estrogen and progesterone receptors and human epidermal growth factor 2 receptor (HER2). This subtype is known for its heterogeneity, immunogenicity and aggressive biological behavior, high metastatic rate, and complex metastatic patterns, altogether leading to a high frequency of relapse and low survival rates^2,3^.

Considering the lack of efficient targeted therapies, chemotherapy is still the most commonly used treatment for TNBC^2,3^. However, chemotherapeutic drugs are well known for being responsible of several adverse effects in patients due to their poor accumulation in the tumor site and metabolization before reaching it, which augment their intrinsic high toxic profile^4^. Other therapeutics as DNA damage agents, angiogenesis inhibitors, anti-androgens and immune checkpoint inhibitors are under clinical evaluation^5,6^.

Edelfosine (1‐O‐octadecyl‐2‐O‐methyl‐sn‐glycero‐3‐phosphocholine or ET) is a synthetic lipid member of alkyl-lysophospholipids family that unlike other chemotherapeutic drugs does not act on the level of DNA. Its high apoptotic action on tumor cells is in part related to its accumulation in their plasma membrane and activation of lipid rafts^7,8^. Edelfosine was tested in phase I^9^ and phase II^10^ trials in patients with acute leukemia, by intravenous and oral administration, showing promising results in purging bone marrow for transplantation. Edelfosine was also tested in a phase II study in patients suffering from brain tumors (inoperable or previously treated with other therapies showing no positive results), by oral administration. The results showed that ET has the capacity to stop brain tumor growth and improve patient’s quality of life^11^. Still, when administered oral or intravenously, this drug leads to gastrointestinal and hemolytic toxicity, respectively^12–14^.

Nanotechnology has already demonstrated its capacity to improve the access of chemotherapeutic drugs to the site of action while decreasing their secondary side effects. In this sense, different authors have proposed ET encapsulation in lipidic nanosystems in order to decrease its related toxicity issues while improving its bioavailability, and therefore its effectiveness on the treatment of different cancers, such as lymphoma^15^, leukemia^16^, osteosarcoma^17^, breast cancer^18^, glioma^19^ as well as related metastasis^20^, among others.

Regarding breast cancer application, Aznar et al. observed a strong inhibition of MCF7 cells (human breast cancer cell line with estrogen and progesterone receptors, i. e. not TNBC-derived) proliferation and a notably decrease in the cell viability upon the treatment with ET lipid nanoparticles in comparison to the free drug. Despite the encouraging results, the performance of this formulation was not studied in vivo*.* In the case of TNBC, considering its high metastatic rate in brain at earlier stages, Ren et al. showed that mice injected with brain metastatic cells derived from TNBC patients, presented a significant inhibition of brain metastatic tumor growth as well as of the formation of macro-metastases upon the treatment with free ET^21^.

Taking this into consideration, in this work we propose the preparation of nanometric emulsions comprising edelfosine (ET-NEs) for the management of TNBC. Unlike the previously mentioned studies that used methods as high shear homogenization and ultrasonication for the preparation of the lipidic nanoparticles, herein we used the simple and mild methodology of ethanol injection, and natural cell components as phosphatidylcholine (PC) and triglycerides as excipients, thereby avoiding the use of surfactants.

ET-NEs’ toxicity was tested both in vitro, using a highly aggressive and invasive TNBC cell line (MDA-MB-231) and in vivo using zebrafish (*Danio rerio*) as animal model. Zebrafish model was selected since it provides the complexity of in vivo conditions that the in vitro cell assays cannot and presents a relevant structural and functional homology to humans with more than 70% of orthologue human genes^22^. Furthermore, in comparison to commonly used rodent animal models it is a more cost-effective and less time-consuming model due to its faster development and small physical size. For instance, basic development is nearly completed within 24 h and sexual maturation is reached in 3–5 months. Nonetheless, one of its main attractive features is the transparent body of zebrafish embryos and larvae, which allows a real-time tracking of the administered or injected fluorescently labeled drugs/nanocarriers and cells of interest^23^.

Regarding the cancer field, different zebrafish cancer models have been developed by transgene expression and xenotransplantation of human tumor cell lines or primary patient-derived cells, among other methods. There are some unique features that make zebrafish an ideal cancer model, being especially relevant its transparency, which as mentioned above allows to monitor tumor development and metastases formation and spreading, as well as their response to treatments^24–26^. Additionally, the delay in the adaptive immune system development (10–14 days) poses a clear advantage over rodent models, as the immunosuppression of animals is not required^27^. It is also worth noting that neovascularization can be also studied due to the similarity between zebrafish and humans’ vasculature^28^. Just as importantly, according to the European Food Safety Administration, zebrafish up to 5 days post-fertilization (dpf) are less prone to experience any pain thereby complying with ethical considerations on animal experimentation (3R principle)^29^. For these reasons, zebrafish cancer models are gaining relevance and different research groups are using them for the evaluation of a number of drugs^30–32^ as well as nanomedicines^33–35^, often as complementary models to the murine ones. On the other hand, Nadar et al., used zebrafish MDA-MB-231 xenograft model for testing their platinum/hydroxyapatite nanoparticles-based therapy against bone cancer, considering the high rate of this type of breast to metastasize into bone^36^.

Following this line, in the present work we decided to determine the efficacy of the developed nanosystem in vitro and in vivo in a zebrafish TNBC model to study the antitumoral potential of the proposed edelfosine nanosystems.

## Results and discussion

### Development and characterization of the NEs

**Figure 1:**
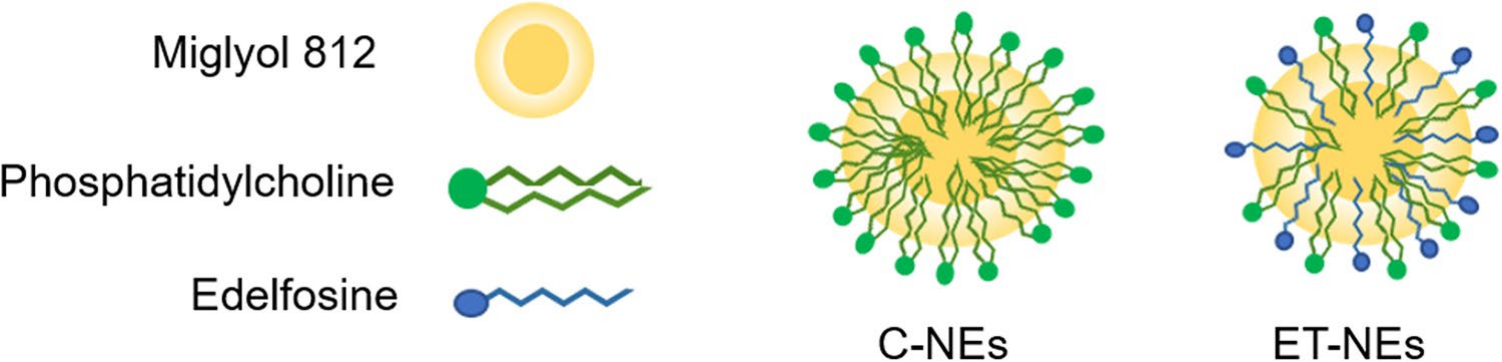
Schematic representation of control (C-NEs) and edelfosine (ET-NEs) nanosystems.

ET-NEs, and their control formulation (C-NEs) were prepared by adapting the ethanol injection method previously optimized by our group for the preparation of nanometric emulsions on a single step^37,38^, allowing the straightforward formation of the nanosystems, as represented in Fig. 1. Edelfosine is composed by a long carbon chain, and phosphate and quaternary amine groups. Considering its lipophilicity, it is expected that it can form emulsions upon combination with an oil (Miglyol) and an additional phospholipid (PC). While the carbon chain can be incorporated into the oily core of the structure, the phosphate and quaternary amine groups can get exposed on its surface, similarly to PC. Indeed, ET was efficiently formulated following this process. ET-NEs composed of 85, 10.7 and 4.3% of Miglyol, ET and PC, respectively, presented a small particle size, a monodisperse population, and a neutral zeta potential (Table 1). The control formulation (C-NEs) was also prepared by replacing ET for PC, presenting a final composition of 85 and 15% of the excipients Miglyol and PC, respectively, which resulted in a similar neutral zeta potential in comparison to ET-NEs and a slightly smaller average size of 100 nm (Table 1).

**Table 1:** Physicochemical properties of edelfosine nanoemulsions (ET-NEs) and the control formulations (C-NEs).

Nanosystem	Size (nm)	PDI	Zeta potential (mV)
C-NEs	131 ± 3	0.1	− 2 ± 0
ET-NEs	123 ± 13	0.1	− 1 ± 0

The nanocarriers properties are influenced by the nanocarrier composition, encapsulated drug, as well as the formulation technique and used solvents. For instance, using a different proportion of lecithin (a type of PC)/Miglyol for indomethacin encapsulation, acetone as solvent, as well as the pouring method, led to the formation of particles of about 220 nm^39^. Eskandar et al. also prepared lecithin/Miglyol particles of about 213 nm (for trans-retinol delivery) by using high pressure homogenization without any organic solvent^40^. On the other hand, the addition of cationic surfactants as hexadecyltrimethylammonium bromide (CTAB) to Miglyol-lecithin by Teijeiro et al. led to the formation of nanocarriers with similar size and polydispersity to our nanosystem, but with a positive net charge instead of neutral/negative one, which in that work was required for the coating with hyaluronic acid^34^. The resulting physicochemical characteristics obtained for the ET formulation herein developed are of particular interest when intravenous administration is envisioned, since particles of this size (< 200 nm) are able to passively enter into the tumor by the enhanced permeability and retention (EPR) effect^41^ and are less prone to accumulate in the liver and spleen, improving their accumulation in the tumor^42^. On the other hand, the neutral zeta potential makes these nanocarriers less prone to opsonization^43^ maintaining their physicochemical properties while circulating in the blood and extending their circulation time in the body. In addition, it makes the nanocarriers more biocompatible in comparison to positively charged ones which interact indiscriminately with cells by electrostatic interactions^44,45^^.^

Aside from the physicochemical properties of the developed nanocarrier, its simple and safe composition, based on GRAS (Generally Recognized as Safe by the United States FDA) materials (Miglyol) and cell components (PC) makes it suitable for the intended application. In addition, the simple and straightforward methodology used for its preparation makes it more advantageous in terms of a pharmaceutical industry point of view, than other lipid nanocarriers reported on literature for ET delivery, which were prepared by hot and high shear homogenization followed by ultrasonication^17–19^. With respect to the concentration of ET in the suspension, we reported about 119 µg ET/mg formulation (theoretical concentration of ET, calculated as µg ET/(mg Miglyol + mg PC)) while other works have reported concentrations of 13 to 33 µg ET/mg formulation^15,16,18,19^.

**Figure 2:**
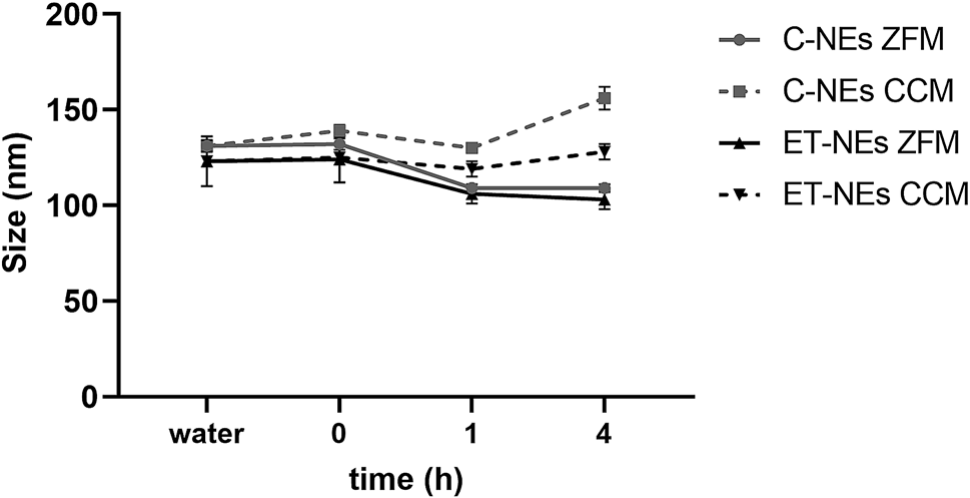
Stability of control NEs (C-NEs) and edelfosine NEs (ET-NEs) upon incubation with zebrafish medium (ZFM, sterile dechlorinated tap water) and cell culture medium (CCM, DMEM supplemented with 1% FBS) for 0, 1 and 4 h, at 37 °C.

In order to determine the feasibility of these formulations for their testing in vitro, ET-NEs as well as the control formulation for reference (C-NEs), were incubated in cell culture media (DMEM with 1% FBS) at 37 ºC, and their particle size was determined for a period of 4 h (Fig. 2). As formulations showed a good stability and maintained their size, we proceeded with their cytotoxic profile evaluation in vitro in MDA-MB-231 cells.

### In vitro studies in TNBC cell line

Taking into account ET related adverse effects and its lipophilicity, different authors have proposed the use of lipid-based nanoparticles for ET delivery^15–17,19^. Blanco-Prieto group has showed that lipid nanosystems composed of Compritol or Precirol and polysorbate 80, prepared by hot and high shear homogenization combined with ultrasonication, are able to decrease ET hematopoietic toxicity, and are also responsible for the improved drug antitumoral efficacy either in vitro and in vivo in glioma, lymphoma and leukemia mice tumor models^15,16,19^. More recently this group has combined ET with doxorubicin^17,20^, and with gemcitabine-squalenic acid conjugates^46^ for osteosarcoma and related metastasis. This group has also tested the effect of ET lipid particles in vitro in the breast cancer cell line MCF7^18^. Nonetheless, to the best of our knowledge, ET has not been tested for TNBC. Considering the aggressiveness of this type of tumor and the antitumoral efficacy of ET in other cancers, we decided to study its effect on TNBC by combining it with our nanosystem prepared by using the simple injection method, as previously mentioned.

**Figure 3:**
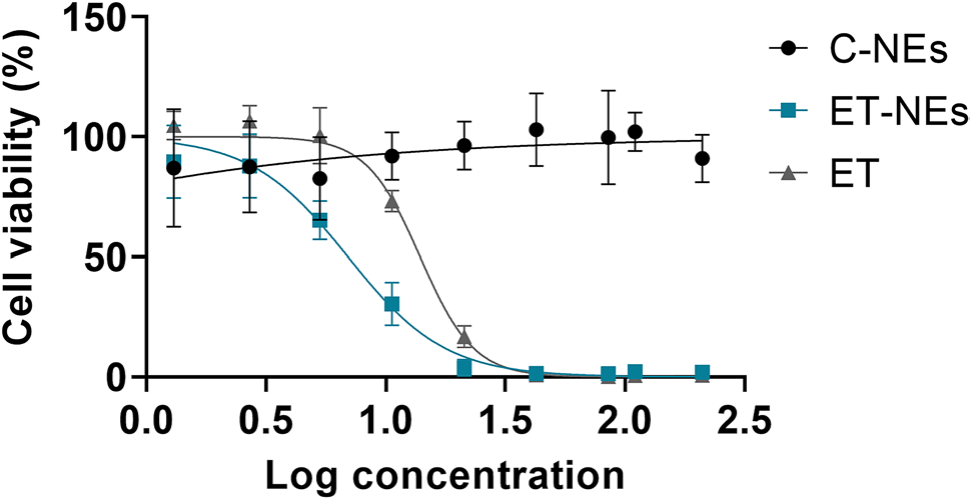
MDA-MB-231 cell viability upon the treatment with increasing concentrations of free edelfosine (ET) edelfosine nanoemulsions (ET-NEs) and control nanoemulsions (C-NEs) during 24 h at 37 °C.

In the present study, ET-NEs and C-NEs were incubated with MDA-MB-231 cells for 24 h, at increasing concentrations of ET (1.3 to 210 µg/mL corresponding to 12.5 to 2000 µg/mL of NEs). As it possible to observe in Fig. 3, and as expected, the control formulation C-NEs do not show cytotoxic effects at the tested range. This is due to the careful selection of the excipients, Miglyol, a GRAS medium chain triglyceride commonly used in self-emulsifying systems, and PC, a major constituent of cell membranes that is present in different nanoformulation compositions. With respect to our formulation ET-NEs, its antitumoral efficacy was dose-dependent, presenting a half maximal inhibitory concentration (IC50) of 6.9 µg/mL (13.2 µM) after 24 h of incubation, while the IC50 of free ET was an order of magnitude higher (13.9 µg/mL, 26.5 µM).

As presented in Table 2, we observed the same tendency in a lung adenocarcinoma (H460) cell line as well as in pancreatic adenocarcinoma cell line (MIA PaCa-2) and pancreatic cells representing liver metastasis (L3.pl6). These cancer cell lines were selected for being representative of prevalent and difficult to treat tumors. As expected, the one presenting a higher resistance for treatment, requiring a higher ET-NEs dose, was our main target cell line MDA-MB-231 (Fig. 3).

**Table 2:** Half maximal inhibitory concentration (IC50) of ET-NEs and free ET upon incubation with different cancer cell lines (lung (H460), pancreatic (MIA PaCa-2), pancreatic liver metastasis (L3.pl6)) for 24 h at 37 °C.

Nanosystem (µg/ml)	MDA-MB-231	H460	MIA PaCa-2	L3.pl6
ET-NEs	6.9	2.2	1.2	2.5
Free ET	13.9	6.1	3.0	5.9

A dose- and time-dependent effect was observed by other authors in human breast cancer (MCF7) and leukemia cell lines with Precirol-polysorbate 80 lipid nanoparticles^16,18^. Our results showed a higher potential of ET for killing a resistant breast cancer cell line in relation to the results reported by Aznar et al. in non-resistant MCF7 breast cancer cells (IC50 12.9 µg/mL after a longer incubation time of 72 h)^18^. Results related to leukemia cell lines reported IC50 of 20 µM in the resistant cell line K562, after 72 h treatment, and IC50 of 3.4 µM for the sensitive cell line MOLM-13^16^. Altogether, and considering the variability due to cell lines tested, we can conclude ET can be efficiently delivered to cancer cells upon formulation as ET-NEs, providing higher efficiencies than the drug in solution (Table 2), and a superior behavior in relation to other nanosystems that have been tested for longer periods of time^16,18^.

**Figure 4:**
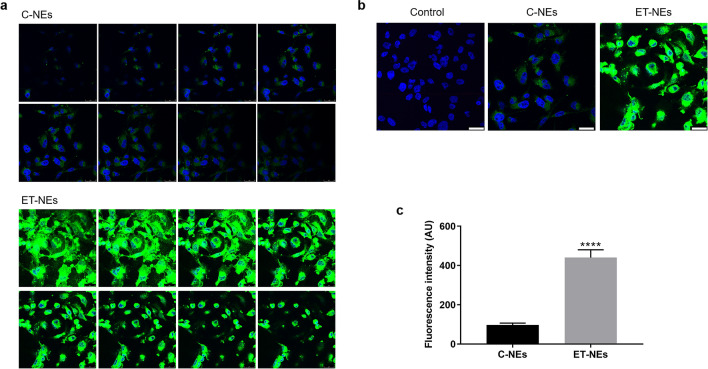
Internalization of NEs in MDA-MB 231 cells. (**a**) Gallery of confocal microscopy images of MDA-MB 231 cells incubated with 150 µg/mL of C-NEs and ET-NEs labeled with TopFluor-PC (green channel) for 4 h at 37 ºC. (**b**) Representative confocal microscopy single-plan image of control vs C-NEs and ET-NEs internalization by MDA-MB 231 cells. (**c**) Fluorescence intensity (AU, arbitrary units) of two maximum projection images (resultant from the combination of all the sections shown in (**a**)) was determined using ImageJ software. Statistical analysis was performed using t test. *P* value ****p < 0.0001. Cell nuclei was stained with Hoechst (blue channel). Scale bars correspond to 25 µm.

Confocal experiments additionally confirmed that ET-NEs were efficiently and highly internalized by the MDA-MB-231, when compared to the C-NEs. Figure 4 presents a gallery showing several sections to show that differences are evident all through the sample (Fig. 4a) as well as a representative single plan for all the samples (Fig. 4b). The green staining that is observed in cells treated with ET-NEs corresponds to the labelled formulation (ET-NEs include TopFluor-PC in a similar amount as in the control formulation). The higher capacity of ET-NEs (*p* < 0.0001) to be internalized (Fig. 4c) might be explained by the well-described characteristic of ET to accumulate in cells’ plasma membrane^7,8^, which herein provided ET-NEs the capacity to be highly internalized.

### In vivo studies in zebrafish embryos

Being aware that the in vitro scenario does not reflect the complexity found in vivo, next step in our research aimed to determine the toxicity, biodistribution, and antitumoral efficacy, of the ET-NEs in vivo*,* using for that purpose zebrafish embryos. This animal model represents a step further to understand tumor heterogeneity, tumor cells behavior and different mechanistic aspects of cancer. Due to its unique characteristics, it is considered a promising tool in the development of novel therapeutic strategies^30,47^.

Prior to test these formulations in vivo, ET-NEs and C-NEs were incubated in SDT water at 28 ºC, and their particle size determined for a period of 4 h, in order to ensure that there were no changes on NEs’ physicochemical properties that could compromise the assay and lead to a misinterpretation of the results. Both formulations were able to maintain their size (Fig. 2), therefore we proceeded with their in vivo evaluation in zebrafish embryos.

## Toxicity assays

### Toxicity of nanosystems upon incubation with 0–4 hpf and 72 hpf zebrafish

Zebrafish embryos were incubated with increasing concentrations (up to 1000 µg/mL) of NEs with and without edelfosine. Above this concentration the media became whiter and the embryos could not be clearly observed through the microscope, therefore we did not test higher concentrations. In accordance to what was previously observed in vitro, at the tested concentrations, C-NEs did not lead to the death of 0 and 72 hpf zebrafish embryos, 96 h post-treatment, irrespectively of the incubation temperature.

In the case of the ET-NEs, the LC50 of the zebrafish maintained at 28 ºC was 12.89 µg/mL (0 hpf) and 8.6 µg/m (72 hpf), while the ones maintained at 34 ºC presented a LC50 of 11.4 µg/mL (0 hpf) and 3.2 µg/mL (72 hpf). The variations in toxicity data seem to be due to the incubation temperatures used in the experiment, as temperature is a relevant and highly variable abiotic factor in nature that plays an important role during fish embryonic development^48^. Furthermore, as temperature increases, it can become a physical stressor and raise the aquatic organisms’ energy metabolism and in turn, the bioavailability of toxicants^49^.

Aside from LC50, other toxicity indexes as LC10, LOEC and NOEC are presented in Table 3. All the requirements of FET test were accomplished: the mortality in negative control embryos was ≤ 10%, the hatching rate was ≥ 80% for the negative control, whereas the positive control was 100% deaths (minimum required is 30%).

**Table 3:** Toxicity of 0 and 72 hpf embryos exposed to ET-NEs for 96 h.

hpf	T (ºC)	LC10	LC50	NOEC	LOEC
0	28	9.8	12.89	10	5
	34	8.6	11.4	10	5
72	28	4.4	8.6	5	1
	34	1.5	3.2	5	1

hpf: hour post- fertilization; LC10, LC50, NOEC and LOEC are represented in µg/mL.

There were no significant morphological changes or hatching delay in comparison with the control group, and following the OECD guidelines, abnormal or non-hatched embryos at 96 hpf were excluded from the assay.

In general, the results show that ET-NEs are clearly more toxic in contrast to the control formulation (C-NEs), a fact that was expected due to the careful selection of the control formulation composition (Miglyol and PC). In specific, Miglyol, a GRAS medium chain triglyceride (used in self-emulsifying systems), and PC, a major constituent of cell membranes (also present in different nanoformulations). These results are in the same line as the in vitro assays, showing a high compatibility of the C-NEs and a dose-dependent toxicity of the ET-NEs.

Taking into consideration the results of the toxicity assay (Table 3), ET-NEs at 1.5 µg/mL (LC10) was selected for further studying the antitumoral efficacy of this nanosystem.

### Toxicity of nanosystems upon injection in 48 hpf zebrafish

Zebrafish embryos of 48 hpf were injected with 5 μg/ml of C-NEs and ET-NEs in the yolk sac or caudal vein and incubated at 28 ºC. As expected, no death was observed 96 h after treatment with any of the tested formulations. These results assure that there would be no deaths as a result of the injection itself combined with the tested nanosystems concentration.

### Biodistribution studies

One of the greatest advantages of zebrafish model organism is that it offers the possibility of studying and fast tracking the distribution of nanoparticles throughout of the organism due to its transparency. In addition, it allows a direct observation of nanoparticles circulation and their interaction with cells^33–35^. In this kind of study, choosing the right dye and the appropriate controls is of extreme importance in order to be sure that we are tracking the nanosystems indeed and avoid the misinterpretation of biodistribution data. On one hand we selected DiR, which is commonly used in biodistribution assays and is easily encapsulated in the nanosystem’s oil core. Nonetheless, it can be released from the oil core of nanosystems causing an apparent but false cellular uptake. Therefore, we also used TopFluor, covalently linked to the PC, and herein anchored to the membrane of the NEs, to help us ensuring that the obtained signal was due to the presence of the NEs by DiR/TopFluor-PC co-localization, and not due to the presence of free fluorophores.

Embryos of 72 hpf were incubated with 500 µg/mL of DiR and TopFluor-PC labeled C-NEs to study its internalization and distribution. This high concentration was necessary to be able to observe the NEs with enough amount of fluorescent labeling, under the confocal microscope. As mentioned before, C-NEs were highly compatible even at the maximum tested concentration of 1000 µg/mL (LC50 could not be assessed), whereas the determined LC50 of ET-NEs was 8.6 µg/mL. Hence, at the necessary concentration (500 µg/mL) for the biodistribution assay, ET-NEs would be lethal to the embryos, which lead us to perform the study only with the control blank formulation (C-NEs).

**Figure 5:**
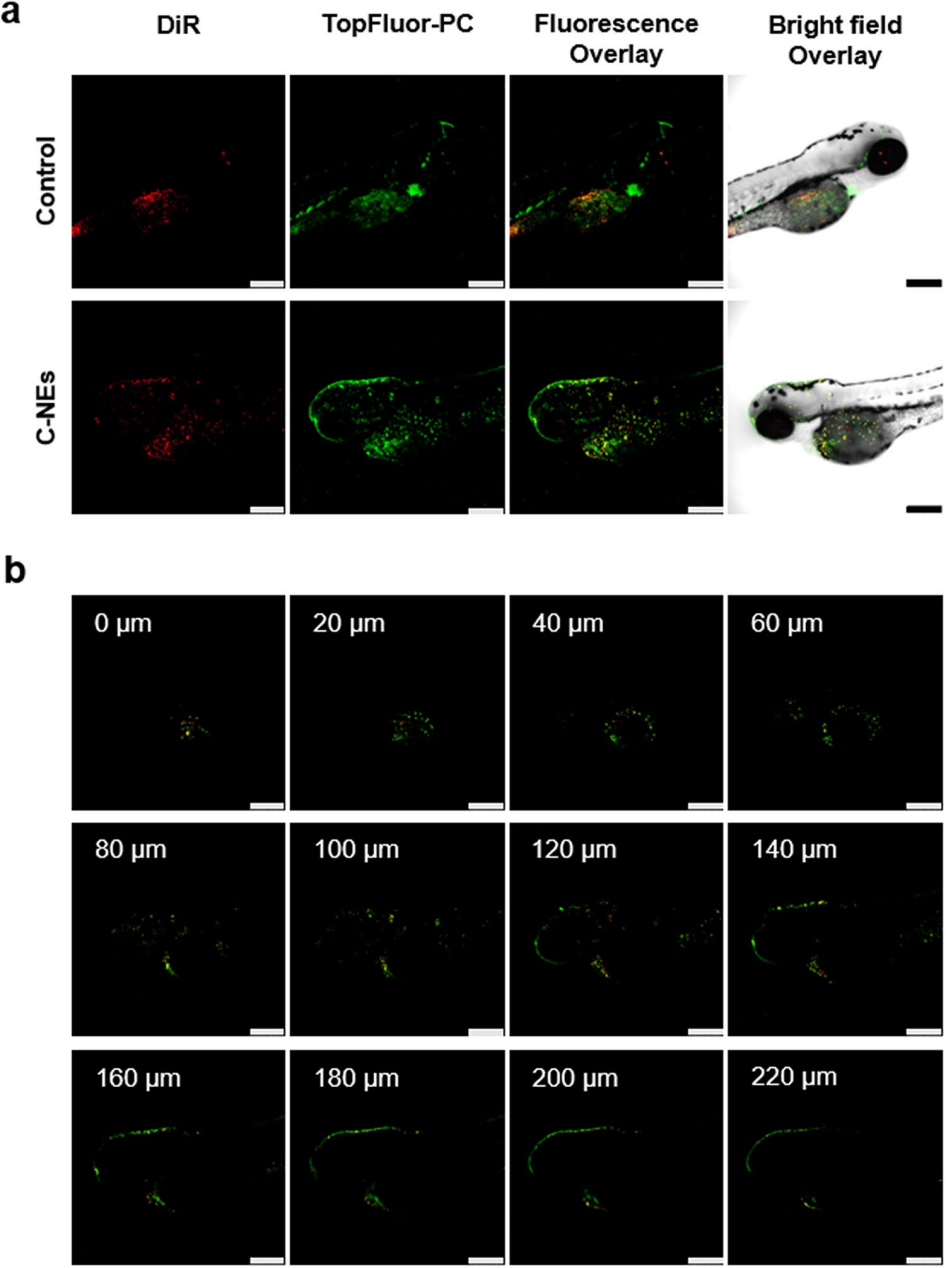
Confocal microscopy images of 72 hpf zebrafish embryos (**a**) incubated with 500 µg/mL of C-NEs labeled with DiR and TopFluor-PC, for 4 h at 34 °C. Control refers to non-treated zebrafish, which present auto-fluorescence. Scale bars correspond to 250 µm. (**b**) Z-stack images. Red channel: DiR. Green channel: TopFluor-PC.

Confocal microscope analysis showed that NEs were efficiently internalized by the exposed embryos, especially into the yolk sac (Fig. 5), which demonstrates the capacity of the C-NEs to cross biological barriers upon direct contact with the skin of zebrafish embryos (without chorion). Free DiR and TopFluor-PC (Fig. 5a control) were just included for reference and not for comparative purposes. Zebrafish of 48 hpf are protected by the chorion. After this period of time, the zebrafish loses this layer (hatching) and the skin becomes the main biological barrier protecting the embryo from the external environment. As indicated, in this study we incubated the NEs with 72 hpf embryo meaning that the NEs were in direct contact with the embryo skin. Teijeiro-Valiño et al. found important differences between the zebrafish biological barriers (chorion vs skin) in terms of the permeability and toxicity of positively charged NEs and negatively charged nanocapsules (NCs)^34^. The NCs containing an external shell of hyaluronic acid/protamine had the capacity to permeate through the chorion and skin layers, unlike the NCs containing only a hyaluronic shell that remained associated to the external layer of these barriers being unable to penetrate. According to the authors the penetration ability of NCs containing protamine could be due to protamine, which aside from its net positive charge is known as a cell penetrating peptide, but could be also due to the presence of PEG-stearate.

Herein the biodistribution of the ET-NEs, under the established conditions i. e. the required concentration for confocal microscopy analysis (500 µg/mL), was not studied considering their higher toxicity than the C-NEs. Unlike negatively charged nanocarriers developed by Teijeiro-Valiño et al., our nanosystems present a neutral surface charge and therefore we hypothesized that C-NEs capacity to penetrate through the zebrafish skin might be due to the presence of PC, which is a major constituent of cell membranes. In addition, taking into consideration the in vitro internalization results in MDA-MB 321 cells, a similar and even greater penetration capacity would be expected for ET-NEs.

### Antitumoral Efficacy of ET-NEs in zebrafish embryos xenotransplanted with TNBC cells

Aside from zebrafish transparency, the possibility to transplant human cancer cells into this animal model is also useful for determining the efficacy of an anti-cancer therapy. In this sense, several models have been developed, leading to a better understanding of different critical aspects of cancer such as proliferation and invasion, tumor formation, angiogenesis, metastasis or immune cell response^50–55^. Furthermore, the tumor microenvironment is more accurately represented than in in vitro models, and the interaction of tumor cells with the host can be inferred, for instance, from the immune cells’ behavior, due to the conserved cell intercommunication between humans and zebrafish^56^. These features, together with those previously mentioned, make the zebrafish a highly valuable platform not only to unravel tumor behavior, but also to test different potential anti-cancer drugs and to perform high-throughput screenings of novel therapeutic compounds. Overall, this brings researchers closer to understanding patients’ response to treatment and, in turn, to personalized medicine^57^.

In this work, our objective was to determine the potential of anti-tumor activity of ET-NEs on zebrafish embryos transplanted with MDA-MB-231 cells (expressing GFP). The yolk sac model is a widely accepted model for drug efficacy studies^58,59^ and herein it was selected in order to avoid modification of the inherited phenotype of the injected cells^52,60,61^ (MDA-MB-231) and to reduce space limitations for cell proliferation^62^.

Stoletov et al. transplanted zebrafish embryos with different breast cancer cell lines and showed that MDA-MB-231 and MDA-MB-435 cells present a high rate of tumor formation, vessel density and metastatic behavior in contrast with low aggressive breast cancers as BT-474^63^. In the same line, Asokan et al. observed that after MDA-MB-231 cells injection into the zebrafish blood circulation, the cells proliferated mainly in the adjacent areas but about 30% presented a metastatic profile^64^. Similarly, Mercatali et al. showed the dissemination and colonization of different zebrafish tissues after MDA-MB-231 xenograft but conversely, when the non-invasive cell line MCF7 was injected, a migratory phenotype was not observed. Additionally, after injecting breast cancer patient-derived bone metastatic primary cells they observed bone marrow tropism, as cells were able to migrate to the caudal hematopoietic tissue, resembling the patient´s clinical profile^65^. These studies provided evidence that this model would be reliable and suitable to test the therapeutic efficiency of the developed formulation.

**Figure 6:**
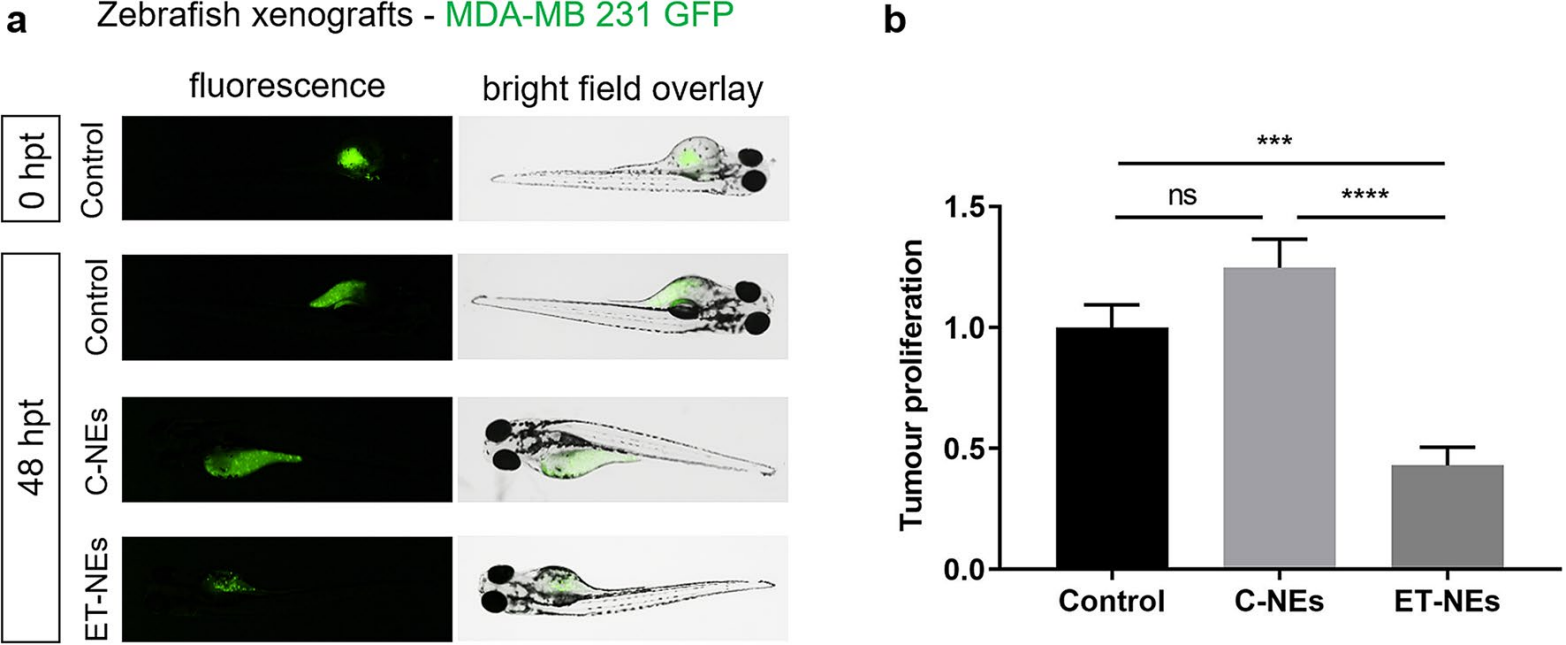
Confocal analysis of tumor cell proliferation in zebrafish (**a**) before (0 hpt) and after 48 hpt. Zebrafish of 48 hpt without chorion were injected with MDA-MB 231 cells expressing GFP and subsequently incubated with ET-NEs and C-NEs at 34 °C. Untreated xenografted embryos were used as control. (**b**) QuantiFish analysis program was used to quantify the fluorescence intensity and determine tumor proliferation. Statistical analysis was performed using One-way ANOVA followed by a Tukey test. *P* values ***p < 0.001, ****p < 0.0001, ns, not significant.

In this sense, at 0 and 48 h post-treatment of the xenotransplated embryos we analyzed the tumor growth by confocal microscopy. As it is possible to observe in Fig. 6a, at 48 hpt there was a reduction of the fluorescence in the yolk sac of the embryos treated with ET-NEs, meaning that there was a reduction in the number of cancer cells, a fact that validated the potential of our approach for the treatment of triple negative breast cancer. As expected, in the case of the control and C-NEs treated embryos, there was an increase of the fluorescent area, indicating tumor growth.

In addition, the analysis performed with QuantiFish program^66^ to quantify the fluorescence intensity and determine the cell growth and spread at the tumor site, corroborates the successful and significant (p < 0.0001) reduction of cell proliferation in xenografted embryos treated with the ET-NEs in comparison with C-NEs treated embryos and the control (Fig. 6b). Other authors have also been able to evaluate different nanocarriers in zebrafish embryos xenografts with important positive results, either by direct injection or incubation according to the size, physicochemical characteristics and properties of the nanoparticles. Evensen et al. used polyethylene glycol-coated nanoparticles in prostate cancer xenografted embryos and demonstrated a low uptake by macrophages and a specific attachment to tumor-like structures^35^. Interestingly, Yang and colleagues developed brain endothelial cell-derived exosomes and used them as vehicle to deliver VEGF siRNAs in a zebrafish xenotransplanted brain tumor model. These natural nanocarriers were able to cross de blood–brain barrier, inhibit VEGF and decrease the fluorescence intensity of cancer cells^67^. Liu et al., used triphenylphosphonium/hyaluronic acid-based nanoparticles bearing doxorubicin in xenotransplanted embryos and observed the inhibition of breast cancer cells proliferation without significant side effects^68^.

With regard to TNBC, so far only Nadar and colleagues have used zebrafish MDA-MB-231 xenograft model in order to determine the anti-tumoral efficacy of platinum-loaded hydroxyapatite nanoparticles. The authors used this animal model since this type of breast tumor frequently metastasize into the bones, which was the main target of the developed therapy. For that, the nanoparticles were co-injected with MDA-MB-231 into the zebrafish blood circulation, a significant (*p* < 0.01) reduction of about 1.9 fold in the number of cancer cells was observed 48 hpi, in comparison to the untreated control. Our nanosystem, composed of the drug edelfosine, with a different mechanism of action (does not act on the DNA level), and formulated as a nanoemulsions making use of GRAS materials, also led to a significant reduction (*p* < 0.001) of tumor proliferation rate of about 2.3 fold in comparison to the untreated control. To the best of our knowledge, it is the first time that the antitumoral efficiency of edelfosine, formulated as an emulsion following a simple methodology and a safe-by-design approach, is studied in TNBC using zebrafish xenograft as animal model. We proved that ET-NEs can be efficiency internalized by cancer cells, leading to a therapeutic effect, in vitro and i*n vivo* (Fig. 6).

## Conclusions

ET-NEs were successfully formulated by ethanol injection, presenting suitable physicochemical properties. The higher toxicity of ET-NEs compared to free ET provides evidence that the ET antitumoral capacity is improved in a dose-dependent manner, in vitro. In addition, in vivo results proved that the nanosystems can penetrate through biological barriers, especially the ones containing edelfosine, which significantly decreased the proliferation of tumoral cells in zebrafish embryos bearing MDA-MB-231 xenograft tumor, leading to its regression. Overall, the obtained results indicate that the developed formulation, ET-NEs, is a promising therapy for the treatment of TNBC.

## Methods

### Materials

Miglyol 812 (CAS 37332–31-3) and phosphatidylcholine (PC, CAS 8002–43-5) were purchased from Lipoid Ludwigshafen, Germany. Edelfosine (CAS 77286–66-9) was acquired from Santa Cruz Biotechnology. DiR lipophilic cyanine dye (CAS 100068–60-8) was supplied from Thermo Fisher Scientific, and TopFluor-PC (CAS 1246355–63-4) from Avanti Polar Lipids. Ethanol (high purity) was obtained from PanReac AppliChem. Dulbecco's Modified Eagle Medium (DMEM) was obtained Sigma, RPMI 1640 Medium, Fetal Bovine Serum (FBS) and penicillin–streptomycin from Thermo Fisher Scientific.

### Preparation and characterization of nanoemulsions

Edelfosine nanoemulsions (ET-NEs) composed by Miglyol 812, PC and ET were formulated by adapting the ethanol injection method^37,69^. In brief, 4 mg of Miglyol, 0.2 mg of PC and 0.5 mg of ET were dissolved in 100 µL of ethanol. ET-NEs were instantaneously formed by injecting the organic phase into 1 mL of ultrapure water under magnetic stirring, at room temperature (RT). ET-NEs were left under magnetic stirring for 10 min to ensure they were completely formed. Control NEs (C-NEs) were prepared by the same method with 4 mg of Miglyol and 0.7 mg of PC.

The particle size, polydispersity index (PDI), and zeta potential of ET-NEs and C-NEs were measured by Dynamic Light Scattering and Laser Doppler Anemometry, using a Zetasizer NanoZS (Malvern Instruments Ltd., UK). Samples were diluted to 1:10 with ultrapure water and the measurements were performed at 25 ºC with a detection angle of 173º. All data is expressed as a mean value ± standard deviation (SD).

### Stability in relevant biological media

The colloidal stability of ET-NEs and C-NEs was evaluated in relevant biological media, to ensure that the formulations maintained their properties during the in vitro and in vivo testing. The formulations with/without ET were incubated with DMEM supplemented with 1% of FBS and with sterile dechlorinated tap (SDT) water at 37 and 28 ºC, respectively, under constant horizontal shaking. Particle size was measured up to 4 h. In all cases, formulations were diluted 1:10 (v/v).

### Preparation of DiR and TopFluor-PC labelled NEs

Fluorescent labelled NE were prepared as previously described in section “Preparation and characterization of nanoemulsions”. Briefly, for the in vitro assays TopFluor-PC (2 µg) was mixed with the other compounds of the organic phase (final volume of 100 µL), while for the in vivo assays, 5 and 10 µg of DiR and TopFluor-PC, respectively, were used. The organic phase was then injected in 1 mL of milli-Q water and kept under magnetic stirring at room temperature for ten minutes.

### In vitro studies

Triple negative breast adenocarcinoma cells MDA-MB-231 (ATCC HTB-26), and pancreatic carcinoma MIA PaCa-2 (ATCC CRL-1420), L3.pl6 (CVCL_0384) were cultured in DMEM, while lung cancer cell line H460 (ATCC HTB-177) was cultured in RPMI at 37 ºC in a humidified atmosphere of 95% air and 5% CO_2_. All cell culture medium (CCM) were supplemented with 10% FBS and 1% penicillin–streptomycin. All cell lines were passed before reaching 80% confluence, 2–3 times a week, and the culture media was replaced every second day.

Twenty thousand MDA-MB-231 cells and 10,000 cells of MIA PaCa-2, L3.pl6 or H460 (cells/well were seeded in 96-well plates and allowed to adhere and grown overnight at 37 ºC and 5% CO_2_. Afterwards, media was replaced with 100 µL of CCM and 25 µL of free ET (stock solution in ethanol and further diluted in water), ET-NEs and C-NEs (previously diluted in water) at increasing concentrations (1.3 to 210 µg/mL of ET corresponding to 12.5 to 2000 µg/mL of NEs) with 6 wells/condition and left incubating at 37 °C for 24 h. Additionally, 25 µL of PBS with 1% Triton X-100 were added as positive control and 25µL of milli-Q water as negative control. Afterwards, cells were washed with PBS, and cell viability was determined by incubating the cells with 110 mL of a filtered MTT solution (0.5 mg/mL in non-supplemented DMEM) at 37 ºC. After 3 h, the solution was removed and the formazan crystals were solubilized with 110 mL of DMSO (15 min at 37 °C, protecting from light). MTT reduction was determined by measuring the light absorbance at 570 nm in a microplate spectrophotometer (DTX 880 Multimode Detector, Beckman Coulter). Cell viability was calculated in percentage related to untreated control wells, subtracting background values.

### Internalization studies

C-NEs and ET-NEs were labeled with Top-Fluor-PC as previously described. In order to evaluate their capacity to be internalized by MDA-MB 231 cells, 80,000 cells were seeded over a glass coverslip in a 24-well plate. After 24 h at 37 ºC, the cells were washed with PBS and afterwards incubated with the nanosystems at a concentration of 150 µg/mL for 4 h at 37 ºC. After, cells were washed twice with PBS, fixed for 15 min at RT with 4% (w/v) paraformaldehyde and washed twice again with PBS. Cell nuclei was stained with Hoechst for 5 min and then cells were washed three times with PBS. The coverslips where then mounted over microscope slides and left to dry overnight, protected from light. Fluorescence intensity of TopFluor-PC (present in C-NEs and ET-NEs) on maximum projection images was determined using ImageJ software. Fluorescence intensity of the control was not determined since it only presents fluorescent cell nuclei (Hoechst) and no TopFluor-PC died cytoplasm.

### In vivo studies in zebrafish

One-year-old adult wild-type zebrafish were maintained in a controlled aquatic facility with purified and dechlorinated water by a reverse osmosis system, with the following conditions: 27 ºC (± 1 ºC), pH 7 (± 0.5), 14/10 h light/dark photoperiod and conductivity 650 µS/cm in 30 L aquaria at a rate of one fish per liter of water. Zebrafish embryos were obtained from mating adults according to previously described procedures^70^. The embryos were collected and washed with osmosis water in Petri dishes and 0–4 h post-fertilization (hpf) embryos were selected with an inverted optical microscope (Nikon TMS). All procedures herein described were approved by the Bioethics Committee for animal experimentation of the University of Santiago de Compostela (CEEA-LU) and were performed in agreement with the standard protocols of Spain (Directive 2012–63-UE) and following the ARRIVE guidelines.

### Acute toxicity assay in zebrafish embryos

Acute toxicity study of ET-NEs and C-NEs was carried out in zebrafish embryos, using the official *Fish Embryo Acute Toxicity* (FET) *test* (Organization for Economic Cooperation and Development, OECD, guideline Test No.236). Previously selected embryos of 0–4 hpf were plated in 96-well plate with 200 µL/well of ET-NEs and C-NEs at different concentrations in SDT water and incubated at 28 °C and 34 °C. These two different temperatures were tested considering that 28 ºC is the temperature usually used for the maintenance of fish while 34 °C is within the temperature range commonly described for xenografts in zebrafish^71^.

Following the mentioned OECD guideline, three replicates of 20 embryos per concentration were used, as well as 24 embryos in separate plates as a negative control, being incubated only in SDT water. Furthermore, in order to determine if plate conditions could lead to alterations during the incubation, internal plate controls were also used, so 10 embryos in SDT water were placed in the same 96-well plates of the experimental conditions. As positive control, 20 embryos were placed in a 24-well plate with a fixed concentration of 4 mg/L 3–4 dichloroaniline and 4 more embryos in SDT water as internal control. Embryos were observed under inverted optical microscope (Nikon TMS) every 24 h until 96 h post-treatment in order to analyze any development alterations, malformations, effects on hatching rate and mortality. On the other hand, in order to study the nanosystems toxicity in hatched embryos (72 hpf), a modification of the FET test was performed, maintaining the experimental conditions, controls and incubation temperature mentioned above.

Statistical analysis of the acute toxicity results was performed using probit analyses with the ToxRat program (ToxRat Solutions. 2003. ToxRat Software for the statistical analysis of biotests. Alsdorf, Germany) in accordance with OECD, guideline 236. Lethal concentration 10 and 50 (LC10 and LC50) were determined using linear maximum likelihood regression with 95% confidence limits, while the lowest observed effect concentration (LOEC) and no-observable-effect concentration (NOEC) were calculated through qualitative trend analysis by contrasts, step-down Cochran-Armitage test and Tarone’s test with 95% confidence limits.

In accordance with the OECD, guideline 236, tests were considered valid if the mortality of embryos in the negative control was less than 10% and more than 30% in the positive control at the end of the 96 h exposure, and the hatching rate of non-treated 0–4 hpf embryos was above 80%.

### Toxicity assay by injection

Toxicity assay by injection was carried out in 48 hpf embryos without chorion. Embryos were anesthetized with 0.003% tricaine (CAS 886–86-2) from Sigma and injected using a borosilicate glass capillary needle (1 mm O.D. × 0.58 mm I.D.; Harvard apparatus) controlled with IM-31 Electric Microinjector (Narishige) with an output pressure of 34 kPa and 25 ms injection time. Five µg/mL of ET-NEs and C-NEs were injected in the yolk or the caudal vein. The embryos were incubated at 28 ºC and evaluated under inverted optical microscope at 24, 48, 72 and 96 h post-injection (hpi) in order to analyze development alterations, malformations, and mortality. Three replicates of 20 embryos were used for each experiment.

### Biodistribution assay

Wild type 72 hpf embryos were incubated with 500 µg/mL of DiR-loaded and TopFluor-PC C-NEs and embryo media (as a control condition) during 4 h at 34 °C, in 24-well plates. Afterwards, the embryos were washed with PBS and fixed with formaldehyde overnight at 4 ºC. Later, they were washed again with PBS and maintained at 4 ºC before visualization. For sample preparation, a Fluorodish (World Precision Instruments, Sarasota, FL, USA) was covered with a layer of agar gel (1% w/v in distilled water) and the zebrafish embryos were placed on top of it. The embryos were observed using a Confocal Microscope Leica TCS SP8 with a HC PL Apo 10x/0.4 objective, and scanned every 10 µm acquiring a total of 27 planes in z-axis direction with a 7.5 × magnification. Images were analyzed using Leica Application Suite X software.

### Xenografts in embryo zebrafish

Zebrafish embryos of 48 hpf, without chorion, were anesthetized with 0.003% tricaine. At least 40 embryos per condition were injected with MDA-MB-231 tumor cells expressing green fluorescent protein (GFP). Initially, MDA-MB-231 cells were trypsinized and 10^6^ cells were resuspended in 10 μl of PBS with 2% polyvinylpyrrolidone (CAS 9003–39-8) from Sigma. The cell suspension was loaded into a glass capillary (1 mm O.D. × 0.58 mm I.D.; Harvard apparatus) and manually injected into the yolk sac of the embryos by electric microinjector with an output pressure of 34 kPa and 30 ms injection time. Embryos which did not present tumor cells inside the yolk sac or that showed cells in circulation after xenotransplantation were considered incorrectly injected and thus, discarded.

The xenotransplanted embryos were incubated with 1.5 µg/ml of ET-NEs or C-NEs at 34 ºC in 24-well plates up to 48 h post-treatment (hpt) and untreated xenografted embryos were used as control. The embryos were photographed at 0 and 48 hpt, using an AZ-100 Nikon fluorescence stereomicroscope and QuantiFish analysis program^66^ was used to quantify the fluorescence intensity, in order to track tumor growth and cell spread in the different treatment conditions.

### Statistical analysis

Unless indicated, all experiments were carried out in triplicate and the results are as presented as mean ± SD. Statistical analysis was performed using GraphPad Prism Software, Inc.Version 8.0.

## References

[1] Khazaei Z (2019). Global cancer statistics 2018: GLOBOCAN estimates of incidence and mortality worldwide stomach cancers and their relationship with the human development index (HDI). World Cancer Res. J..

[2] Harbeck, N. *et al.* Breast cancer. *Nat. Rev. Dis. Primers* **5**, 10.1038/s41572-019-0111-2 (2019).

[3] Garrido-Castro AC, Lin NU, Polyak K (2019). Insights into molecular classifications of triple-negative breast cancer: improving patient selection for treatment. Cancer Discov..

[4] Church D (2014). 'Toxgnostics': an unmet need in cancer medicine. Nat. Rev. Cancer.

[5] Malla RR (2019). A perspective on the diagnostics, prognostics, and therapeutics of microRNAs of triple-negative breast cancer. Biophys. Rev..

[6] Bianchini G, Balko JM, Mayer IA, Sanders ME, Gianni L (2016). Triple-negative breast cancer: challenges and opportunities of a heterogeneous disease. Nat. Rev. Clin. Oncol..

[7] Mollinedo F, Gajate C, Martín-Santamaría S, Gago F (2004). ET-18-OCH3 (edelfosine): a selective antitumour lipid targeting apoptosis through intracellular activation of Fas/CD95 death receptor. Curr. Med. Chem..

[8] Gajate C, Mollinedo F (2014). Lipid rafts, endoplasmic reticulum and mitochondria in the antitumor action of the alkylphospholipid analog edelfosine. Anticancer Agents Med. Chem..

[9] Berdel WE, Fink U, Rastetter J (1987). Clinical phase I pilot study of the alkyl lysophospholipid derivative ET-18-OCH3. Lipids.

[10] Vogler, W. R. *et al.* in *Platelet-Activating Factor and Related Lipid Mediators 2* 389–396 (Springer, 1996).

[11] Nagler, A. Edelfosin for the treatment of brain tumors. (2003).

[12] Lasa-Saracíbar B (2014). Lipid nanoparticles protect from edelfosine toxicity in vivo. Int. J. Pharm..

[13] Briglia M, Fazio A, Signoretto E, Faggio C, Lang F (2015). Edelfosine induced suicidal death of human erythrocytes. Cell. Physiol. Biochem..

[14] de AlmeidaPachioni J (2013). Alkylphospholipids–a promising class of chemotherapeutic agents with a broad pharmacological spectrum. J. Pharmacy Pharmaceutical Sci..

[15] de Mendoza AE-H (2012). Complete inhibition of extranodal dissemination of lymphoma by edelfosine-loaded lipid nanoparticles. Nanomedicine.

[16] Lasa-Saracíbar B, de Mendoza AE-H, Mollinedo F, Odero MD, Blanco-Príeto MJ (2013). Edelfosine lipid nanosystems overcome drug resistance in leukemic cell lines. Cancer Lett..

[17] González-Fernández Y (2017). Doxorubicin and edelfosine lipid nanoparticles are effective acting synergistically against drug-resistant osteosarcoma cancer cells. Cancer Lett..

[18] Aznar MÁ, Lasa-Saracíbar B, de Mendoza AE-H, Blanco-Prieto MJ (2013). Efficacy of edelfosine lipid nanoparticles in breast cancer cells. Int. J. Pharm..

[19] de Mendoza AE-H, Préat V, Mollinedo F, Blanco-Prieto MJ (2011). In vitro and in vivo efficacy of edelfosine-loaded lipid nanoparticles against glioma. J. Control. Release.

[20] González-Fernández Y, Brown HK, Patiño-García A, Heymann D, Blanco-Prieto MJ (2018). Oral administration of edelfosine encapsulated lipid nanoparticles causes regression of lung metastases in pre-clinical models of osteosarcoma. Cancer Lett..

[21] Ren D (2018). Targeting brain-adaptive cancer stem cells prohibits brain metastatic colonization of triple-negative breast cancer. Can. Res..

[22] Howe K (2013). The zebrafish reference genome sequence and its relationship to the human genome. Nat. Biotech..

[23] Sieber S (2019). Zebrafish as a preclinical in vivo screening model for nanomedicines. Adv. Drug Deliv. Rev..

[24] Gutierrez-Lovera C, Vazquez-Rios AJ, Guerra-Varela J, Sanchez L, de la Fuente M (2017). The potential of zebrafish as a model organism for improving the translation of genetic anticancer nanomedicines. Genes.

[25] Stoletov K, Klemke R (2008). Catch of the day: zebrafish as a human cancer model. Oncogene.

[26] Blackburn JS, Langenau DM (2014). Zebrafish as a model to assess cancer heterogeneity, progression and relapse. Dis. Model. Mech..

[27] Lam S, Chua H, Gong Z, Lam T, Sin Y (2004). Development and maturation of the immune system in zebrafish, Danio rerio: a gene expression profiling, in situ hybridization and immunological study. Dev Comput. Immunol..

[28] Zhao S, Huang J, Ye J (2015). A fresh look at zebrafish from the perspective of cancer research. J. Exp. Clin. Cancer Res..

[29] Authority EFS (2005). Opinion of the scientific panel on animal health and welfare (AHAW) on a request from the commission related to the aspects of the biology and welfare of animals used for experimental and other scientific purposes. EFSA J..

[30] MacRae CA, Peterson RT (2015). Zebrafish as tools for drug discovery. Nat. Rev. Drug Discovery.

[31] Lenis-Rojas OA (2017). Dinuclear RuII (bipy) 2 derivatives: Structural, biological, and in vivo zebrafish toxicity evaluation. Inorg Chem.

[32] Penas C (2016). Light-controlled cellular internalization and cytotoxicity of nucleic acid-binding agents: studies in vitro and in zebrafish embryos. ChemBioChem.

[33] Crecente-Campo J (2019). The size and composition of polymeric nanocapsules dictate their interaction with macrophages and biodistribution in zebrafish. J. Control. Release.

[34] Teijeiro-Valiño C (2017). Assessment of the permeability and toxicity of polymeric nanocapsules using the zebrafish model. Nanomedicine.

[35] Evensen L (2016). Zebrafish as a model system for characterization of nanoparticles against cancer. Nanoscale.

[36] Nadar RA (2020). Preclinical evaluation of platinum-loaded hydroxyapatite nanoparticles in an embryonic zebrafish xenograft model. Nanoscale.

[37] Bouzo BL, Calvelo M, Martin-Pastor M, Garcia-Fandino R, de la Fuente M (2020). In vitro-in silico modelling approach to rationally design simple and versatile drug delivery systems. J. Phys. Chem. B.

[38] Nagachinta S, Bouzo BL, Vazquez-Rios AJ, Lopez R, de la Fuente M (2020). Sphingomyelin-based nanosystems (SNs) for the development of anticancer miRNA therapeutics. Pharmaceutics.

[39] Calvo P, Vila-Jato JL, Alonso MJ (1996). Comparative in vitro evaluation of several colloidal systems, nanoparticles, nanocapsules, and nanoemulsions, as ocular drug carriers. J. Pharm. Sci..

[40] Eskandar NG, Simovic S, Prestidge CA (2009). Chemical stability and phase distribution of all-trans-retinol in nanoparticle-coated emulsions. Int. J. Pharm..

[41] Peer D (2007). Nanocarriers as an emerging platform for cancer therapy. Nat. Nanotechnol..

[42] Blanco E, Shen H, Ferrari M (2015). Principles of nanoparticle design for overcoming biological barriers to drug delivery. Nat. Biotechnol..

[43] Aggarwal P, Hall JB, McLeland CB, Dobrovolskaia MA, McNeil SE (2009). Nanoparticle interaction with plasma proteins as it relates to particle biodistribution, biocompatibility and therapeutic efficacy. Adv. Drug Deliv. Rev..

[44] Arvizo RR (2011). Modulating pharmacokinetics, tumor uptake and biodistribution by engineered nanoparticles. PLoS ONE.

[45] Asati A, Santra S, Kaittanis C, Perez JM (2010). Surface-charge-dependent cell localization and cytotoxicity of cerium oxide nanoparticles. ACS Nano.

[46] Rodríguez-Nogales C (2019). A unique multidrug nanomedicine made of squalenoyl-gemcitabine and alkyl-lysophospholipid edelfosine. Eur. J. Pharm. Biopharm..

[47] Stern HM, Zon LI (2003). Cancer genetics and drug discovery in the zebrafish. Nat. Rev. Cancer.

[48] Radonic M, López AV, Oka M, Aristizábal EO (2005). Effect of the incubation temperature on the embryonic development and hatching time of eggs of the red porgy Pagrus pagrus (Linne, 1758)(Pisces: Sparidae). Rev. Biol. Mar. Oceanogr..

[49] Heugens EH, Hendriks AJ, Dekker T, Straalen NM, Admiraal W (2001). A review of the effects of multiple stressors on aquatic organisms and analysis of uncertainty factors for use in risk assessment. Crit. Rev. Toxicol..

[50] Naber HP, Drabsch Y, Snaar-Jagalska BE, ten Dijke P, van Laar T (2013). Snail and Slug, key regulators of TGF-β-induced EMT, are sufficient for the induction of single-cell invasion. Biochem. Biophys. Res. Commun..

[51] Yang X-J (2013). A novel zebrafish xenotransplantation model for study of glioma stem cell invasion. PLoS ONE.

[52] Haldi M, Ton C, Seng WL, McGrath P (2006). Human melanoma cells transplanted into zebrafish proliferate, migrate, produce melanin, form masses and stimulate angiogenesis in zebrafish. Angiogenesis.

[53] Tobia C, Gariano G, De Sena G, Presta M (2013). Zebrafish embryo as a tool to study tumor/endothelial cell cross-talk. Biochimica et Biophysica Acta -Molecular Basis of Disease.

[54] Stoletov K, Montel V, Lester RD, Gonias SL, Klemke R (2007). High-resolution imaging of the dynamic tumor cell–vascular interface in transparent zebrafish. Proc. Natl. Acad. Sci..

[55] Zhang B, Xuan C, Ji Y, Zhang W, Wang D (2015). Zebrafish xenotransplantation as a tool for in vivo cancer study. J. Familial Cancer.

[56] Tulotta, C. *et al.* in *Zebrafish* 155–169 (Springer, 2016).

[57] Costa B, Estrada MF, Mendes RV, Fior R (2020). Zebrafish avatars towards personalized medicine—a comparative review between avatar models. Cells.

[58] Lin H-S (2019). Identification of novel anti-liver cancer small molecules with better therapeutic index than sorafenib via zebrafish drug screening platform. Cancers.

[59] Wrobel JK (2020). Rapid in vivo validation of HDAC inhibitor-based treatments in neuroblastoma zebrafish xenografts. Pharmaceuticals.

[60] Vargas-Patron, L. A. *et al.* Xenotransplantation of Human glioblastoma in Zebrafish larvae: in vivo imaging and proliferation assessment. *Biology open* **8** (2019). 10.1242/bio.043257 PMC6550087 31085547

[61] Lee LM, Seftor EA, Bonde G, Cornell RA, Hendrix MJ (2005). The fate of human malignant melanoma cells transplanted into zebrafish embryos: assessment of migration and cell division in the absence of tumor formation. Dev. Dyn..

[62] Letrado P, de Miguel I, Lamberto I, Díez-Martínez R, Oyarzabal J (2018). Zebrafish: speeding up the cancer drug discovery process. Can. Res..

[63] Stoletov K (2010). Visualizing extravasation dynamics of metastatic tumor cells. J. Cell Sci..

[64] Asokan, N. *et al.* Continuous high-resolution in vivo imaging reveals tumor-specific dissemination in an embryonic zebrafish xenograft model. *bioRxiv*, 215921 (2017).

[65] Mercatali L (2016). Development of a patient-derived xenograft (PDX) of breast cancer bone metastasis in a zebrafish model. Int. J. Mol. Sci..

[66] QuantiFish - A zebrafish fluorescence analyser v. 1.1 (Zenodo, 2017).

[67] Yang T (2017). Delivery of small interfering RNA to inhibit vascular endothelial growth factor in zebrafish using natural brain endothelia cell-secreted exosome nanovesicles for the treatment of brain cancer. AAPS J..

[68] Liu H-N (2018). Delivery of mitochondriotropic doxorubicin derivatives using self-assembling hyaluronic acid nanocarriers in doxorubicin-resistant breast cancer. Acta Pharmacol. Sin..

[69] Jaafar-Maalej C, Diab R, Andrieu V, Elaissari A, Fessi H (2010). Ethanol injection method for hydrophilic and lipophilic drug-loaded liposome preparation. J. Liposome Res..

[70] Westerfield M (2000). The zebrafish book: a guide for the laboratory use of zebrafish (Danio rerio).

[71] Konantz M (2012). Zebrafish xenografts as a tool for in vivo studies on human cancer. Ann. NY Acad. Sci..

